# Exploring healthcare experiences of transgender people in the Jabula Uzibone study, South Africa: a longitudinal implementation science study

**DOI:** 10.1002/jia2.26503

**Published:** 2025-07-07

**Authors:** Rutendo Bothma, Audrey Pettifor, Innocent Maphosa, Philisiwe Ndlovu, John Imrie, Tonia Poteat

**Affiliations:** ^1^ Wits RHI University of the Witwatersrand Johannesburg South Africa; ^2^ Department of Epidemiology Gillings School of Public Health University of North Carolina at Chapel Hill Chapel Hill North Carolina USA; ^3^ Division of Epidemiology and Biostatistics Department of Global Health Faculty of Medicine and Health Sciences Stellenbosch University Stellenbosch South Africa; ^4^ Division of Healthcare in Adult Populations Duke University School of Nursing Durham North Carolina USA

**Keywords:** differentiated service delivery, gender‐affirming care, healthcare access, South Africa, stigma, transgender healthcare

## Abstract

**Introduction:**

The World Health Organization promotes a transgender‐differentiated service delivery (TG‐DSD) model to overcome barriers to HIV service engagement among transgender people (TGP). For TGP, an essential element of DSD includes gender‐affirming care which is non‐stigmatising, free from discrimination and celebrates their gender identity. The *Jabula Uzibone* Study, launched in November 2023, assesses the cost and effectiveness of TG‐DSD on HIV outcomes. In this paper, we describe the baseline characteristics of TGP in our study and explore whether there are differences in healthcare experiences among those seeking care at TG‐DSD clinics versus standard service delivery (SSD) clinics at baseline.

**Methods:**

This observational, mixed‐method, prospective implementation study compares models of care at four TG‐DSD and four SSD facilities using standardised observation checklists, in‐depth and key informant interviews. For this paper, we asked participants about healthcare experiences and experiences of stigma through a structured, interviewer‐administered quantitative survey. We assessed the sections of the quantitative survey which ask about self‐reported experiences of stigma.

**Results:**

The study enrolled 422 TGP with HIV (217 TG‐DSD and 205 SSD) and 248 TGP without HIV (128 TG‐DSD and 120 SSD); 15% (102/670) gender non‐conforming, 15% (91/670) TG men and 70% (477/670) TG women. Participants’ median age was 29 years, interquartile range: 24−35 years. SSD participants at baseline were 46% more likely to experience stigma compared to their TG‐DSD counterparts (aOR = 1.46, 95% CI: 1.06, 2.01). SSD participants were more likely to encounter a healthcare provider who is unwilling to provide care for them (aOR = 1.55, 95% CI: 1.09, 2.21) and to report that healthcare workers are unable to provide the same quality care to TGP as they do other people (aOR = 1.46, 95% CI: 1.00, 1.91) compared to their TG‐DSD counterparts.

**Conclusions:**

TGP from TG‐DSD facilities were less likely to report experiences of facility‐based enacted stigma at baseline, compared to the TGP from SSD facilities. Our study highlights the importance of provider training in tailored transgender healthcare to provide gender‐affirming healthcare services. Results from the *Jabula Uzibone* study will provide further evidence of the effectiveness of TG‐DSD models in sub‐Saharan Africa, and the role of stigma and discrimination in HIV outcomes among TGP.

## 1 INTRODUCTION

Despite growing global recognition of the unique challenges faced by transgender people (TGP) within health systems, they continue to experience systemic discrimination, a lack of culturally competent care and provider bias. Consequently, TGP experience significant health disparities, including a disproportionately higher prevalence of HIV [1, 2, 3]. Contributing factors include healthcare avoidance, fear of discrimination and previous negative healthcare experiences within healthcare settings [4, 5, 6]. HIV prevalence among transgender women is estimated at 63% in Johannesburg, and approximately 50% in Cape Town and Buffalo City; data for Nelson Mandela Bay were unavailable at the time of writing [7].

In South Africa, recent data from 1423 TGP revealed persistent systemic discrimination in public health facilities. Only 7% of respondents felt safe and comfortable accessing care at public health facilities, and 10% reported being denied care in 2022 due to their gender identity or sexual orientation [8]. Despite constitutional guarantees of the right to health, TGP in South Africa continue to encounter healthcare denial and provider unwillingness to provide care [3, 9]. A systematic review exploring access and utilisation among African transgender communities reported that discriminatory practices among healthcare providers led to shame, fear and anxiety [2]. These experiences contribute to mistrust in the health system and delays in seeking timely medical care, further exacerbating health disparities [10].

To address such inequities, the World Health Organization and the International AIDS Society have endorsed differentiated service delivery (DSD) models which aim to improve access and engagement in care among marginalised populations [11, 12]. For transgender individuals, DSD models are designed to remove barriers such as provider biases, discrimination and a lack of affirming services [13]. Central to transgender‐specific DSD (TG‐DSD) is the provision of gender‐affirming healthcare, which includes the use of chosen names and pronouns, access to psychosocial support, provision of gender‐affirming hormone therapy (GAHT) and the creation of inclusive, discrimination‐free clinical environments [14]. Evidence indicates that TGP who access gender‐affirming healthcare demonstrate improved quality of life, higher engagement in care, better adherence to HIV treatment, improved psychological wellbeing and reduced disparities compared to those without access to such services [15, 16, 17, 18].

While GAHT can be administered safely within a primary healthcare setting, it is seldom offered in public health facilities in sub‐Saharan African countries [19]. Accessing GAHT typically requires regular clinic visits and monitoring, which may foster routine health‐seeking behaviour and improve overall outcomes. In South Africa, TG‐DSD services are primarily delivered through donor‐funded programmes, which are geographically limited. As a result, many TGP must rely on public healthcare facilities where stigma and discrimination from facility staff persists [8, 16]. Wits RHI delivers TG‐DSD models in four South African health districts (Buffalo City, City of Cape Town, Johannesburg and Nelson Mandela Bay) through funding from the United States Agency for International Development (USAID).

Despite well‐documented barriers to healthcare access for TGP, there remains a paucity of research directly comparing service delivery models. The *Jabula Uzibone* study (Zulu term meaning Be Happy and See Yourself) aims to evaluate the effectiveness and cost of TG‐DSD in delivering HIV prevention and care compared to standard service delivery (SSD) clinics, that is public health facilities. The overall study hypothesises that healthcare experiences differ substantially between DSD and SSD settings, driven by the affirming environments at TG‐DSD clinics. This paper describes the baseline characteristics of enrolled participants and presents a cross‐sectional analysis of baseline healthcare experiences in DSD versus standard service delivery (SSD) clinics.

## 2 METHODS

The *Jabula Uzibone* study is an observational multi‐site mixed methods prospective implementation science study assessing the implementation of TG‐DSD models and SSD models at the facility level using standardised observation checklists at all eight facilities (four DSD and four SSD). Participants will complete a quarterly survey to assess changes over time in experiences of gender affirmation as well as experiences of stigma. For TGP on antiretroviral therapy (ART), we will measure their HIV RNA levels at baseline and at 12 months. For TGP on pre‐exposure prophylaxis (PrEP), we will measure tenofovir levels at baseline and at 12 months. The objective of this paper is to assess the baseline characteristics of participants in this study, focusing on the differences between self‐reported healthcare experiences among the TG‐DSD and SSD participants.

### 2.1 Study setting

We conducted the study in the four South African health districts where Wits RHI clinics are located. Buffalo City and Nelson Mandela Bay clinics are co‐located within the department of health facilities in a park home, which serves as a separate clinic from the facility in which they are located. The City of Cape Town and Johannesburg operate stand‐alone clinics. These four clinics, operating since 2019, were the first USAID‐funded transgender clinics in South Africa [16]. Staff provide services to over 5000 TGP in 1 year, more than 1000 are on ART, close to 1000 are on PrEP and more than 600 are on GAHT. Peer educators, who mostly identify as transgender or queer, drive the community outreach model. All sites offer free gender‐affirming care, which includes GAHT, and other primary healthcare services. The SSD facilities, operated by the Department of Health in South Africa, provide standard primary healthcare, including HIV prevention and treatment service, but exclude GAHT.

### 2.2 Participant eligibility, recruitment, enrolment and data collection

#### 2.2.1 Eligibility

Eligibility criteria included: being ≥ 18 years old, having a gender identity that differs from sex assigned at birth, receiving healthcare in one of the TG‐DSD clinics or in an SSD clinic within the four districts. Participants who tested HIV negative may or may not be on oral PrEP, and participants who tested HIV positive were required to have been prescribed ART.

#### 2.2.2 Recruitment

We used stratified purposive sampling to ensure the enrolment of participants. Peer educators, who all identified as transgender or queer, recruited participants from their social networks, via social media posts, WhatsApp groups, and word of mouth in the community and facilities within the study districts using flyers and palm cards. Power statistics and sample size for the overall study were based on the primary outcome of viral suppression. There are no documented viral suppression results for TGP, nevertheless, we used 47% as reported by Fearon et al. [20]. Our unpublished study among TGP in South Africa showed that those who were on GAHT were 2.73 times more likely to be virally suppressed compared to TGP who did not have access to GAHT, suggesting an effect size ∼20%. An effect size as low as 15% is considered clinically significant. We considered our sample size based on these data and an estimated loss to follow‐up of ∼10%, to provide adequate statistical power. Once in the study, participants could switch their model of care dependent on service convenience, as it would have been unethical to restrict participants from choosing their preferred model of service delivery.

#### 2.2.3 Enrolment

Peer educators pre‐screened participants who met the eligibility criteria and conducted a rapid HIV test to confirm self‐reported HIV status. Study staff conducted the informed consent process in private areas, ensuring that the potential participants understood all the study requirements, including the potential risks of study participation. Once the participants consented to participate, they signed the informed consent form, and the study staff offered them a copy to take home. Enrolment commenced in November 2023 and the final participant was enrolled in September 2024.

#### 2.2.4 Data collection

Data collection took place either in the Wits RHI TGD‐DSD clinics or in the community (in a private gazebo, a dedicated consulting room at an SSD facility or in a mobile clinic). Study staff administered a 1‐hour long electronic quantitative survey for participants. Socio‐demographic information including age, education, gender and gender identity was collected. We operationalised gender based on participant self‐report as gender non‐conforming, transgender man and transgender woman.

We assessed the sections of the quantitative survey which asks about self‐reported experiences of stigma for the objective of this paper. Enacted stigma can be described as outright discrimination, violence or derision of people's experience because of an actual or perceived transgender identity [21, 22]. Stigma was assessed using the brief health facility stigma index developed by Nyblade et al. [23]. The index includes four items with Yes/No response options (e.g. Has the following happened to you or others when getting healthcare—“Healthcare workers unwilling to care for a transgender person or gender non‐conforming client?”). A response of “yes” was coded as 1 and a “no” was a 0. Then, scores were summed for each participant to arrive at a final score. Higher scores indicate greater facility‐based stigma. Responses of “don't know,” or “prefer not to answer” were treated as missing for the final analysis. For the four questions, the range of this type of response was from 1.94% to 4.93%.

We also assessed healthcare experiences by asking participants about their experiences when they accessed healthcare in the past 3 months (e.g. “Did a healthcare provider refuse to give you care?”), with possible responses of “yes,” “no,” “don't know” and “prefer not to answer.” Participants who responded, “don't know,” or “prefer not to answer” were excluded from the final analysis (range: 0.15−1.02%). We also read out statements to participants as if it were a statement they made and asked them to rate each one with “never,” “rarely,” “sometimes,” “often,” “always,” “don't know,” “does not apply” and “prefer not to answer.” The statement included items such as “I delay seeking medical care because of my gender identity or expression.” Participants who responded, “don't know,” “does not apply” or “prefer not to answer” were excluded from the final analysis (0.3−8.8%).

### 2.3 Data analysis

We used STATA 18.0 [24] to conduct statistical analysis for the baseline results. Categorical variables, such as healthcare experiences, were described with proportions, and continuous variables, such as age, were presented as medians with standard deviations and interquartile ranges. We performed chi‐square tests for associations between categorical variables. We used Pearson's correlations for bivariate analyses of continuous variables. Variables significant at *p*‐value < 0.2 on univariate analysis were included in the multivariable analyses to compare differences in various experiences based on participants accessing care at a TG‐DSD or SSD site.

### 2.4 Ethical considerations

The *Jabula Uzibone* study was approved by the University of Witwatersrand Human Research Ethics Committee (Protocol Number: M220420) and the Duke University Health System Institutional Review Board (Protocol Number:00113320). Participants signed the informed consent form before any study procedures were conducted. Participants were reimbursed with ZAR300 (USD16) for each study visit.

## 3 RESULTS

### 3.1 Characteristics of enrolled participants

The study enrolled 670 transgender participants across the four sites (Table 1). The majority, 71% (477/670), were transgender women, 15% (102/670) were gender non‐conforming and 14% (91/670) were transgender men. The median age of the participants was 29 years, with an interquartile range of 24−35 years, and the majority, 64% (429/670), completed high school. Most of the participants were from Johannesburg, 41% (275/670) across both models. Of the 248 participants without HIV, 56% (138/248) reported to have taken oral PrEP to prevent HIV, and 73% (101/139) had taken oral PrEP in the last 30 days. Forty percent of participants (263/654) reported they wanted to be on hormone therapy, but they did not have access to it. Participant's gender, age, PrEP and GAHT use were significantly associated (*p* < 0.01) with the service delivery model.

**Table 1:** Characteristics of *Jabula Uzibone* participants

	Overall *N* (%)	Transgender‐differentiated service delivery *n* (%)	Standard service delivery *n* (%)	*p*‐value
**Gender**				
Gender non‐conforming	102 (15.22)	48 (47.1)	54 (52.9)	0.005
Transgender man	91 (13.58)	34 (37.4)	57 (62.6)	
Transgender woman	477 (71.19)	263 (55.1)	214 (44.9)	
**Age**				
**Median, interquartile range**		28 (24−34)	30 (25−36)	
18−24	196 (29.25)	114 (58.2)	82 (41.8)	0.051
25−29	174 (25.97)	95 (54.6)	79 (45.4)	
30−34	141 (21.04)	67 (47.5)	74 (52.5)	
35−39	82 (12.24)	37 (45.1)	45 (54.9)	
40+	77 (11.49)	32 (41.6)	45 (58.4)	
**Education**				
Primary	9 (1.34)	3 (33.3)	6 (66.7)	0.1
Grade 8–10	94 (14.03)	48 (51.1)	46 (48.9)	
Grade 11–12	429 (64.03)	211 (49.2)	218 (50.8)	
Higher education	138 (20.60)	83 (60.1)	55 (39.9)	
**District service delivery model**				
Buffalo City Municipality TG‐DSD	48 (7.16)	48 (100)		< 0.001
Buffalo City Municipality SSD	42 (6.27)		42 (100)	
City of Cape Town TG‐DSD	102 (15.22)	102 (100)		
City of Cape Town SSD	101 (15.07)		101 (100)	
Johannesburg TG‐DSD	140 (20.90)	140 (100)		
Johannesburg SSD	134 (20)		134 (100)	
Nelson Mandela Bay TG‐DSD	55 (8.21)	55 (100)		
Nelson Mandela Bay SSD	48 (7.16)		48 (100)	
**HIV status**				
HIV negative	248 (37.01)	128 (51.6)	120 (48.4)	0.96
HIV positive	422 (62.99)	217 (51.4)	205 (48.6)	
**Ever started oral PrEP**				
No	110 (44.35)	36 (33.96)	74 (69.81)	< 0.001
Yes	138 (55.65)	93 (70.45)	45 (34.09	
**Taken oral PrEP in past 30 days**				
No	38 (27.34)	20 (52.6)	18 (47.4)	0.028
Yes	101 (72.66)	73 (72.3)	28 (27.7)	
**Currently on hormone therapy**				
No, and I do not want to be on hormones	184 (28.13)	71 (38.6)	113 (61.4)	< 0.001
Yes	207 (31.65)	150 (72.5)	57 (27.5)	
No, but I want to be taking hormones	263 (40.21)	115 (43.7)	148 (56.3)	

Abbreviations: PrEP, pre‐exposure prophylaxis; SSD, standard service delivery; TG‐DSD, transgender‐specific differentiated service delivery.

### 3.2 Facility‐based enacted stigma

Half (50%, 331/666) of the participants reported that they had experienced at least one of the four manifestations of facility‐based stigma (Table 2). Stigma experiences included healthcare workers unwilling to provide healthcare to TGP because of their gender identity (31%, 200/654); experiencing poorer quality of healthcare being provided to TGP compared to other people (38%, 248/657); having heard healthcare workers talk badly about a transgender person (38%, 243/647); and having a healthcare worker unnecessarily disclose a client's gender identity (32%, 206/637).

**Table 2:** Facility‐based enacted stigma reported by *Jabula Uzibone* participants

	Overall *N* (%)	Transgender‐differentiated service delivery *n* (%)	Standard service delivery *n* (%)	*p*‐value
**Healthcare workers unwilling to care**
Yes	200 (30.58)	90 (45)	110 (55)	0.027
**Healthcare workers providing poorer quality of care**				
Yes	248 (37.75)	115 (46.4)	133 (53.6)	0.043
**Healthcare workers talking badly**				
Yes	243 (37.56)	119 (49)	124 (51)	0.268
**Healthcare workers disclosing a client's gender identity**				
Yes	206 (32.34)	94 (45.6)	112 (54.4)	0.031

### 3.3 Healthcare experiences

More than a quarter (29%, 191/667) of the participants reported that they had ever avoided seeking medical care they needed because of fear of being mistreated as a transgender person (Table 3), 19% (129/667) had done so in the past 12 months. Seventy‐three percent (73%) of DSD and SSD participants had seen a healthcare provider in the past 3 months and reported several bad experiences, including, being disrespected (10%, 47/483), healthcare provider refusing to provide care (5%, 25/487) and being harassed at a healthcare facility while receiving care (8%, 37/485). Additionally, a third (32%, 180/558) of the participants reported at least one negative experience during a healthcare visit.

**Table 3:** Healthcare experiences among *Jabula Uzibone* participants

	Overall *N* (%)	Transgender‐differentiated service delivery *n* (%)	Standard service delivery *n* (%)	*p*‐value
**Ever avoided healthcare because of previous bad experiences**				
No	476 (71.36)	252 (52.9)	224 (47.1)	0.496
Yes, in the past 12 months	129 (19.34)	59 (45.7)	70 (54.3)	
Yes, more than 12 months ago	62 (9.30)	32 (51.6)	30 (48.4)	
**Seen a healthcare provider in the last 3 months**				
Yes	488 (72.94)	266 (54.5)	222 (45.5)	0.02
**Healthcare experiences**				
**Treated with respect**				
Yes	436 (90.27)	242 (55.5)	194 (44.5)	0.094
**Healthcare provider refused to give you care**				
Yes	25 (5.13)	16 (64)	9 (36)	0.344
**Harassed in a healthcare setting**				
Yes	37 (7.63)	20 (54.1)	17 (45.9)	0.412
**Touched inappropriately by someone during a healthcare visit**				
Yes	21 (4.30)	11 (52.4)	10 (47.6)	0.841

### 3.4 Health survey

Forty‐six percent of participants reported having ever delayed seeking medical care because of their gender identity or expression (Table 4). More than a quarter (27%, 176/663) of the participants reported that they are never comfortable seeking healthcare in their own communities and (20%, 134/655) reported that being transgender changed the way healthcare providers interacted with them. Twenty‐three percent (21%, 141/665) of participants reported they are always afraid of being harassed when they seek medical care, and (15%, 97/667) reported that they always accept mistreatment due to their gender identity or expression to get medical care ((Table 5)).

**Table 4:** Health survey results from *Jabula Uzibone* participants

	Overall *N* (%)	Transgender‐differentiated service delivery *n* (%)	Standard service delivery *n* (%)	*p*‐value
**Health survey results**				
**Delay seeking medical care**				
Never	361 (54.20)	199 (55.1)	162 (44.9)	0.312
Rarely	90 (13.51)	41 (45.6)	49 (54.4)	
Sometimes	134 (20.12)	68 (50.7)	66 (49.3)	
Often	44 (6.61)	17 (38.6)	27 (61.4)	
Always	37 (5.56)	18 (48.6)	19 (51.4)	
**Healthcare providers provide same quality of care to transgender people as they do to other peoplea**				
Never	147 (22.51)	87 (59.2)	60 (40.8)	0.033
Rarely	75 (11.49)	41 (54.7)	34 (45.3)	
Sometimes	170 (26.03)	94 (55.3)	76 (44.7)	
Often	95 (14.55)	45 (47.4)	50 (52.6)	
Always	166 (25.42)	69 (41.6)	97 (58.4)	
**Comfortable seeking healthcare in own communitya**				
Never	176 (26.55)	117 (66.5)	59 (33.5)	< 0.001
Rarely	59 (8.90)	35 (59.3)	24 (40.7)	
Sometimes	111 (16.74)	55 (49.5)	56 (50.5)	
Often	74 (11.16)	34 (45.9)	40 (54.1)	
Always	243 (36.65)	99 (40.7)	144 (59.3)	
**Being transgender changes the way healthcare provider interacts with me**				
Never	170 (25.95)	88 (51.8)	82 (48.2)	0.811
Rarely	52 (7.94)	23 (44.2)	29 (55.8)	
Sometimes	208 (31.76)	112 (53.8)	96 (46.2)	
Often	91 (13.89)	47 (51.6)	44 (48.4)	
Always	134 (20.46)	66 (49.3)	68 (50.7)	
**Experience with health providers has been positive**				
Never	18 (2.69)	10 (55.6)	8 (44.4)	0.079
Rarely	37 (5.53)	14 (37.8)	23 (62.2)	
Sometimes	125 (18.68)	54 (43.2)	71 (56.8)	
Often	104 (15.55)	61 (58.7)	43 (41.3)	
Always	385 (57.55)	205 (53.2)	180 (46.8)	
**Experience with mental health providers has been positive**				
Never	21 (3.44)	8 (38.1)	13 (61.9)	0.201
Rarely	30 (4.91)	11 (36.7)	19 (63.3)	
Sometimes	87 (14.24)	42 (48.3)	45 (51.7)	
Often	85 (13.91)	42 (49.4)	43 (50.6)	
Always	388 (63.50)	217 (55.9)	171 (44.1)	
**Afraid of experiencing physical violence/abuse when seeking medical care**				
Never	296 (44.51)	158 (53.4)	138 (46.6)	0.708
Rarely	83 (12.48)	46 (55.4)	37 (44.6)	
Sometimes	121 (18.20)	60 (49.6)	61 (50.4)	
Often	24 (3.61)	13 (54.2)	11 (45.8)	
Always	141 (21.20)	65 (46.1)	76 (53.9)	
**Afraid of experiencing harassment when seeking medical care**				
Never	273 (41.05)	150 (54.9)	123 (45.1)	0.431
Rarely	83 (12.48)	44 (53)	39 (47)	
Sometimes	132 (19.85)	66 (50)	66 (50)	
Often	26 (3.91)	10 (38.5)	16 (61.5)	
Always	151 (22.71)	73 (48.3)	78 (51.7)	
**I have to educate healthcare providers about my healthcare needs**				
Never	163 (24.40)	95 (58.3)	68 (41.7)	0.538
Rarely	68 (10.18)	33 (48.5)	35 (51.5)	
Sometimes	163 (24.40)	82 (50.3)	81 (49.7)	
Often	73 (10.93)	36 (49.3)	37 (50.7)	
Always	201 (30.09)	98 (48.8)	103 (51.2)	
**I accept mistreatment due to my gender identity**				
Never	381 (57.12)	205 (53.8	176 (46.2)	0.074
Rarely	52 (7.80)	17 (32.7	35 (67.3)	
Sometimes	98 (14.69)	56 (57.1	42 (42.9)	
Often	39 (5.85)	17 (43.6	22 (56.4)	
Always	97 (14.54)	49 (50.5	48 (49.5)	

^a^ Number of responses excluded those who answered neutrally.

**Table 5:** Multivariable logistic regression analysis to investigate differences in healthcare experiences among TG‐DSD and SSD participants

	Unadjusted odds ratios (95% confidence interval)	*p‐*value	Adjusted odds ratios (95% confidence interval)^a^	*p‐*value
Healthcare workers unwilling to provide healthcare to a transgender person	1.45 (1.04−2.04)	0.03	1.55 (1.09−2.21)	0.01
Healthcare workers providing poorer quality of healthcare to a transgender person	1.39 (1.01−1.90)	0.04	1.46 (1.00−1.91)	0.03
Healthcare workers talking badly about a transgender person	1.20 (0.87−1.65)	0.27	1.25 (0.89−1.75)	0.19
Healthcare workers disclosing a client's transgender or gender non‐conforming identity with colleagues without consent when not medically necessary	1.44 (1.03−2.01)	0.03	1.45 (1.02−2.05)	0.04
Enacted stigma	1.38 (1.01−1.87)	0.04	1.46 (1.06−2.01)	0.02

Abbreviations: SSD, standard service delivery; TG‐DSD, transgender‐specific differentiated service delivery.

^a^ The multivariable model adjusted for HIV status, age and education.

### 3.5 Examining differences in healthcare between differentiated service delivery model and SSD model

#### 3.5.1 Facility‐based enacted stigma

We examined facility‐based enacted stigma as an overall score and each of the forms of facility‐based enacted stigma (i.e. healthcare workers: 1. unwilling to provide healthcare to a transgender person; 2. providing poorer quality of healthcare to a transgender person; 3. talking badly about a transgender person; 4. disclosing a client's transgender identity with colleagues without consent when not medically necessary). Participants who accessed healthcare services at the SSD facilities were 1.46 times (aOR = 1.46, 95% CI [1.06, 2.01]) more likely to experience facility‐enacted stigma compared to their TG‐DSD counterparts. Additionally, when each of the forms of facility‐enacted stigma were examined for differences between service delivery models adjusting for age, education and receiving hormone therapy, SSD participants were more likely to have a healthcare provider who is unwilling to provide care for them (aOR = 1.55, 95% CI [1.09, 2.21]) and to report poorer quality care from healthcare workers (aOR = 1.46, 95% CI [1.00, 1.91]) compared to TG‐DSD participants. Additionally, SSD participants were 1.45 times more likely to have a healthcare worker disclose their gender identity without consent when not medically necessary (aOR = 1.45, 95% CI [1.02, 2.05]) compared to TG‐DSD participants.

## 4 DISCUSSION

Our findings on facility‐based enacted stigma reveal that TGP continue to face a myriad of negative experiences when they access healthcare, and those who access SSD facilities are more likely to experience stigma. These results echo a systematic review which concluded that TGP in Africa continue to experience healthcare stigma [2]. Gender‐affirming care in these facilities could reduce stigma and potentially lead to improved overall health, psychological wellbeing and self‐fulfilment as reported in other reviews [2, 18].

Participants who accessed SSD facilities were more likely to experience healthcare worker unwillingness to provide care, report poorer quality of care compared to their cisgender counterparts and unnecessary disclosure of their gender identity. This is consistent with some studies where TGP reported unwillingness from healthcare workers to provide care, being denied medication and having their gender identity disclosed without their consent [4, 25, 26]. King et al. noted that transgender participants in their Ugandan study reported receiving inferior healthcare compared to cisgender people [27]. Several studies have documented healthcare stigma and healthcare worker attitudes as a key barrier to healthcare access and utilisation for TGP [28, 29, 30], consistent with our findings.

When asked about their health experience in the last 3 months, a third of the participants reported at least one of the following: avoiding healthcare, being disrespected, refused care, harassed or touched inappropriately at the healthcare facility. Previous research has documented healthcare avoidance by TGP and is consistent with our study findings [5, 6, 31]. Negative experiences such as the ones reported by our participants and lack of adequate gender‐affirming care could drive TGP to avoid healthcare or forego seeking appropriate medical care even when they are in poor health, further exacerbating health inequalities.

Analysis of healthcare experiences by HIV status suggested that differences in healthcare experiences were primarily driven by the model of service delivery rather than the HIV status itself. Specifically, TGP living with HIV from SSD sites were more likely to report healthcare worker unwillingness to provide care and breaches of confidentiality on their gender identity. However, these disparities were not significantly different from those reported by TGP living without HIV from the SSD sites, suggesting that HIV status alone did not drive these negative experiences.

Our study findings reflect the scarcity of GAHT, which TGP often prioritise over other healthcare needs [32], with 31% of participants reporting they had access to GAHT. This is consistent with a review from nine countries which reported that only 31% of participants had access to hormone therapy [2]. In a South African study, 49% of participants expressed the desire to affirm their gender with hormone therapy but only 11% had access to such therapy [33]. Limited access to GAHT among the *Jabula Uzibone* study participants may be because of such therapy only being available at tertiary healthcare institutions in South Africa or financial constraints if TGP resort to private healthcare. Although our study findings did not explore this, studies have documented that TGP often resort to unsupervised and illicit hormone use in the absence of accessible, affordable and affirming care [4, 9].

At least a fifth of our participants reported that healthcare workers do not provide the same quality of care to them as they do cisgender people, and that being transgender changes the way healthcare professionals interact with them. This finding could be a lack of clinical competence among health workers to provide appropriate care for TGP as has been found in several studies [34, 35]. Incorporating specialised curricula for healthcare providers such as sensitisation training which is tailored to the transgender and gender‐diverse communities is an essential intervention to improve trans‐specific knowledge among healthcare providers [2, 36].

We found that 14% of participants had avoided healthcare in the last year, and more than 20% of them were afraid of experiencing violence or being harassed at healthcare facilities. Mbeda et al. [37] reported fear of seeking medical care as the most frequent consequence of healthcare‐related stigma among TGP. In other studies, transgender participants reported discrimination and shaming as drivers of their fear and anxiety when accessing healthcare [26, 38]. The repercussions of discriminatory behaviour amplify health disparities and can threaten the success of health programmes such as HIV prevention and treatment [39, 40].

Our study has limitations. As an observational study, we could not randomise study sites or participants; and doing so may have been seen as unethical due to the well‐documented benefits of gender‐affirming care for TGP. However, enrolling participants from existing TG‐DSD demonstration sites and comparing them with participants who receive care at SSD sites provided a key opportunity to assess TG‐DSD models in real‐world settings. The data presented in this cross‐sectional paper is self‐reported and may be skewed by recall or social desirability bias, or reluctance to divulge sensitive individual experiences to interviewers. To minimise this, the study recruited and trained data collectors to maximise participant comfort, safety and confidentiality.

TGP who seek care at TG‐DSD clinics may have different healthcare‐seeking behaviours, experiences and expectations compared to those who access SSD clinics. As such, they may have been more likely to report positive healthcare experiences compared to their SSD counterparts. In our regression analysis, we controlled for different potential confounders such as HIV status, age and education, to mitigate this bias. Our study could also be limited by selection bias. First, differences in healthcare experiences between TG‐DSD and SSD participants may reflect underlying differences in population characteristics rather than the direct effects of the model of care. Second, the findings may not be fully generalisable to all TGP in South Africa, particularly those who do not access care through either model. To mitigate this selection bias, we endeavoured to enrol a diverse range of participants within each stratum.

## 5 CONCLUSIONS

Our study contributes to the growing literature on healthcare experiences among TGP and fills an important gap in knowledge about healthcare among TGP in South Africa. Our results highlight the critical need for gender‐affirming healthcare in South African health facilities. We have shown that TGP continue to face pervasive stigma, discrimination and negative health experiences when they seek medical care. We noted several significant differences in health experiences between participants who sought care at TG‐DSD versus SSD facilities, with the latter reporting more negative health experiences in some instances. Our findings support the call for clinically and culturally competent healthcare providers in South Africa. Providing gender‐affirming care, including hormone therapy, is imperative, especially at a primary care level where accessibility is easier compared to tertiary or quaternary hospitals, which may require long and/or expensive commutes for TGP.

The National Department of Health in South Africa has developed the Key Populations Health Implementation Plan which seeks to address the health needs of key populations, including TGP [41]. In the health plan, gender‐affirming healthcare for TGP is highlighted as a cornerstone to improving their healthcare. The full rollout of this plan could capacitate healthcare workers with the necessary skills, resources and knowledge to provide gender‐affirming care, thereby reducing incidents of facility‐based stigma among TGP. Further research to understand the readiness of the South African health system to provide gender‐affirming care is indicated.

## COMPETING INTERESTS

The authors declare that they have no competing interests.

## AUTHORS’ CONTRIBUTIONS

TP, RB and AP: Conceptualisation. RB and PN: Data curation. IM and TP: Methodology. RB: Writing—original draft. All authors: Writing—review and editing.

## FUNDING

Research reported in this publication was supported by the United States National Institute of Mental Health of the National Institutes of Health under Award Number R01MH130277. The funder had no role in the conceptualisation, design, data collection, analysis, decision to publish or preparation of this manuscript.

## DISCLAIMER

The content is solely the responsibility of the authors and does not necessarily represent the official views of the United States National Institutes of Health. 

## Data Availability

The datasets generated during the current study will be available from the senior author upon reasonable request 1 year after the completion of data collection.
